# Features of the use of ladder myoplasty of a gunshot wound to the laryngopharynx: Case report

**DOI:** 10.1016/j.ijscr.2023.108875

**Published:** 2023-10-01

**Authors:** I.A. Lurin, V.V. Makarov, E.M. Khoroshun, V.V. Nehoduiko, S.A. Shypilov, K.M. Smolianyk

**Affiliations:** a National Academy of Medical Sciences of Ukraine, Kyiv, Ukraine; b State Scientific Institution "Scientific and Practical Center of Preventive and Clinical Medicine" of the State Administration of Affairs, Kyiv, Ukraine; c Military Medical Clinical Center of the Northern Region, Kharkiv, Ukraine; d Kharkiv National Medical University, Kharkiv, Ukraine

**Keywords:** The laryngopharynx wound, Myoplasty, The sternocleidomastoid muscle, Combat conditions, Case report

## Abstract

**Introduction and importance:**

The laryngopharynx wound is considered to be one of the most severe wounds of neck both in war and in peace, as it may cause life threatening changes in the whole body (asphyxia, bleeding, shock). Important aspects of surgical treatment are to ensure full breathing, acceptable ways of feeding, and the use of reliable wound closure techniques aimed to prevent digestive tract failure and to maintain the framework and aerostasis of the laryngotracheal region.

**Case presentation:**

A case of unilateral multiple wounds of the laryngopharynx was described in the article. The features of diagnostics, surgical treatment and conservative therapy in the postoperative period with this injury were presented. The wounded man was urgently operated. During surgery the pharynx was mobilized. The metal fragment was removed. The wound of the pharynx was sutured with a two-row suture. The next stage of the surgical treatment was myoplasty. In the case of the patient, the purpose of myoplasty was additional sealing of the pharyngeal suture and myoplasty of the thyroid cartilage injury zone for the purpose of aerostasis. Because of the size of the wounds and their anatomical localization, we used the mobilized lower edge of the Musculus sternocleidomastoideus for myoplasty and proposed the method of ladder myoplasty developed by us.

**Clinical discussion:**

In myoplasty method the following criteria must be followed: the muscle flap must be of sufficient length and width, so as not to cause excessive tension in the myoplasty area; the flap must be thick enough to avoid necrosis that may cause subsequent infectious complications; when taking the flap, the most sparing operative access should be used to avoid functional and anatomical disorders; the volume of the taken muscle flap must not lead to functional and anatomical disorders.

**Conclusion:**

The proposed method of ladder myoplasty using Musculus sternocleidomastoideus is unique, and proves its high efficiency in unilateral multiple laryngopharyngeal injuries, and can be recommended for wide clinical implementation in such clinical situations.

## Highlights

• The laryngopharynx wound is considered to be one of the most severe wounds of neck.
• The most consistent with the specified criteria for myoplasty in this area is myoplasty using the sternocleidomastoid muscle.
• The case described by us with the use of ladder myoplasty is presented for the first time in Ukraine.
• The proposed method of ladder myoplasty using the sternocleidomastoid muscle is unique, has shown its high efficiency.

## 1 Introduction

The laryngopharynx wound is considered to be one of the most severe wounds of neck both in war and in peace, as it may cause life threatening changes of wounded people (asphyxia, bleeding, shock). Important aspects of surgical treatment are to ensure full breathing, acceptable ways of feeding, and the use of reliable wound closure techniques aimed to prevent digestive tract failure and to maintain the framework and aerostasis of the laryngotracheal region [[1], [2], [3]].

Foreign and domestic authors note the effectiveness of myoplasty in treatments of wounds of the larynx and pharynx in order to achieve aerostasis and improve the reliability of suturing the digestive tract [[4], [5], [6], [7], [8], [9], [10]].

The use of Musculus sternocleidomastoideus with a single flap is widely described by foreign authors for single wounds of the larynx or pharynx [[11], [12], [13], [14], [15], [16], [17], [18]]. Domestic authors describe the use of myoplasty using opposing flaps of the right and left Musculus sternocleidomastoideus for single injuries of the larynx, cervical trachea using transverse cervical approach [2,7].

The choice of the method of myoplasty for multiple unilateral wounds of the laryngopharynx remains relevant. Any description of the recommended myoplasty technique for these injuries in the available domestic as well as foreign literature sources still remains undiscovered. The following case (described with the use of ladder myoplasty) is presented for the first time in Ukraine against the background of the military aggression of the Russian Federation. The case report is prepared according to the SCARE Criteria [19].

## 2 Presentation of case

A case of unilateral multiple wounds of the laryngopharynx was described in the article. The features of diagnostics, surgical treatment and conservative therapy in the postoperative period with this injury were presented.

Wounded A., aged 27, was admitted 3 h after he had being wounded to the Military Medical Clinical Center of the Northern Region with a blind penetrating wound to the right lateral surface. Upon admission, he complained of pain in the right side of the neck, hoarseness.

**Fig. 1:**
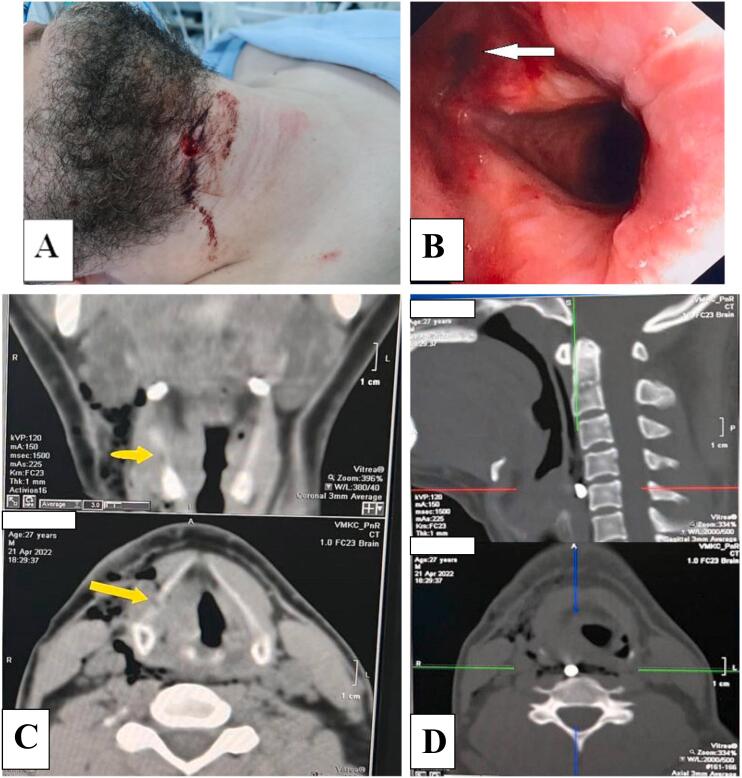
Examination of a Gunshot Wound to the Laryngopharynx (A- view of the wound on admission; B - endoscopic examination - injury of the right lateral surface of the pharynx; C - CT scan - the site of injury of the right half of the thyroid cartilage; D - localization of a foreign body, metal fragment).

At the initial examination, the patient's condition was of moderate severity. The body temperature was 37.1 °C. The skin and mucous membranes were pale pink. External breathing was spontaneous through the nose. The frequency of respiratory movements was 20 in 1 min. Pulse – 92/ min, blood pressure 110/80 mmHg. The inlet of the wound channel was determined along the right side surface up to 7 mm in diameter. On palpation, there was pain on the right side of the neck and in the area of the wound. Puffiness of the soft tissues of the neck was observed, as well as moderate subcutaneous emphysema along the right lateral surface of the neck (Fig. 1A).

Examined by an ENT doctor. Performed bronchoscopy - revealed swelling of the larynx, rigidity of the right vocal cord.

The Computed tomography (CT) revealed the presence of air in the projection of the right half of the pharynx and in the soft tissues of the right lateral surface of the neck. The CT showed a through damage of the right half of the thyroid cartilage. The presence of a metal fragment in the pharyngeal space in the projection of the 4th cervical vertebra (Fig. 1C, D).

The patient underwent endoscopic examination that showed a wound in the right half of the pharynx (Fig. 1B).

The wounded man was urgently operated. General anesthesia with tracheal intubation was applied. The patient was intubated unconscious. Tracheal intubation was performed under bronchoscopy guidance to ensure airway safety. A combined lateral cervical approach was performed (access along the medial part of the right Musculus sternocleidomastoideus and additional access in the submandibular region on the right). The revision revealed a penetrating gunshot wound of the right half of the thyroid cartilage.

**Fig. 2:**
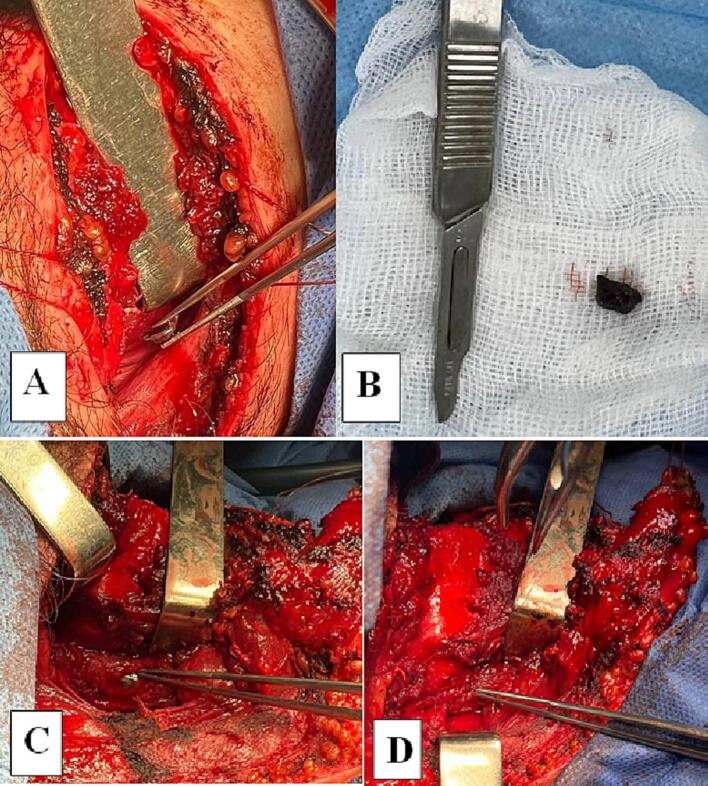
The pharynx wound (A – the removal of a foreign body (the metal fragment) of the pharyngeal space; B – the view of the removed metal fragment; C - the view of the wound of the pharynx; D - the site of the wound of the pharynx after suturing).

During surgery the pharynx was mobilized. The metal fragment was removed. (Fig. 2A, B).

The place of injury of the lateral wall of the pharynx was identified. The edges of the wound were excised. The wound of the pharynx was sutured with a two-row suture (Fig. 2C, D). After that, the pharyngeal space was drained.

The next stage of the surgical treatment was myoplasty. The myoplasty case was applied with a strand of the right Musculus sternocleidomastoideus measuring 15 × 7 cm. For this type of myoplasty, a sample of ½ muscle strand was taken according to the recommendations of Ukrainian authors [6,7]. The stated sample is sufficient for effective closure of the wounded area and does not lead to dysfunction of the remaining muscle.

**Fig. 3:**
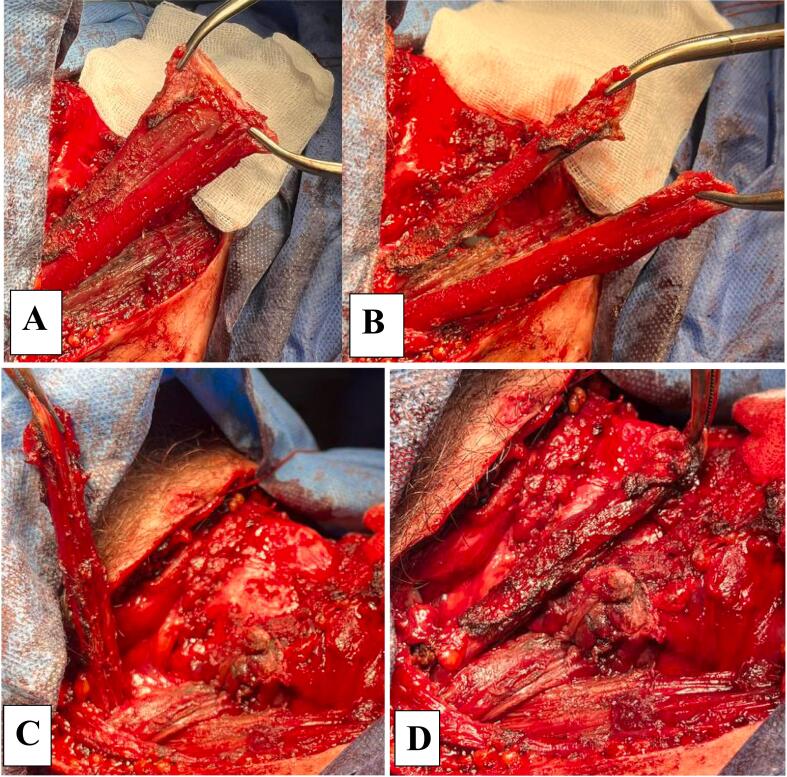
Ladder myoplasty (A – the mobilization of the muscle strand for myoplasty, B – the dissection of the flap for the formation of steps, C – the formation of a short muscle step, D – the formation of a long muscle step).

The peculiarity of the proposed method of myoplasty is that after the isolation of the muscle flap, it was divided in this case into two separate flaps in order to subsequently form muscle strands (steps). When forming muscle steps for myoplasty, it is possible to dissect the flap in a ratio of 1:1 or 1:2, the main criteria for this division are the viability of the flaps and the size of the zones of wound defects requiring myoplasty [2,7]. In this case, we used a 1:1 flap dissection (Fig. 3A, B).

When forming muscle steps in scalariform myoplasty, it is important to avoid crossing the muscle steps at the base of the muscle flap. It is necessary to perform ladder myoplasty consistently, the first stage is to form a short step - myoplasty of the area of the nearest injury is performed. In this case, it was the area of suturing the pharynx. After that, we excised the excess part of the first muscle step. When forming muscle steps, we consider it mandatory to excise a portion of the muscle (rotational platform), on which either a clamp was applied or sutured with a thread and the muscle flap was rotated in order to avoid necrosis of this portion of the flap in the future. The second stage was myoplasty itself – it was performed in a long step. In this case, it was myoplasty of the wounded thyroid cartilage. The peculiarity of the wound of the thyroid cartilage was that the skeletal function had been preserved, myoplasty was required to achieve aerostasis. An important point in performing myoplasty was the fixation of muscle flaps to the wounded area. The muscle flap was sutured only from the side of the wound adjacent to the area with vicryl No. 3. Flashing through the muscle flap could lead to its necrosis in the future. During the formation of the short step, the muscle flap was sutured to the pharynx in the area of its suturing, the long step was sutured to the perichondrium of the thyroid cartilage. The area of the rotation platform of the long step was also excised (Fig. 3C, D).

The surgical wound was sutured in layers with the installation of drainage. The patient also underwent a lower tracheostomy and gastrostomy by Kader. The oropharyngeal cavity was tamponed with gauze swabs.

**Fig. 4:**
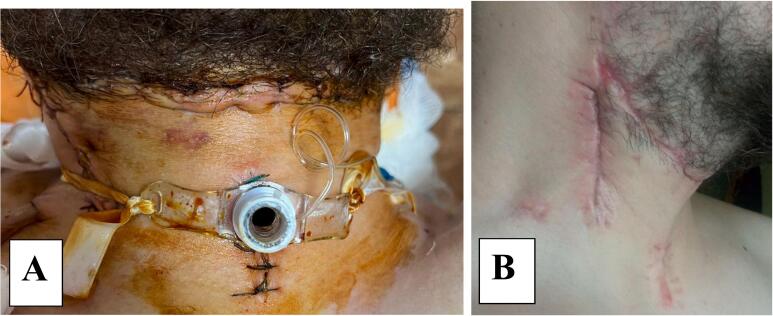
View of the patient's postoperative neck (A - wound on the second day of the postoperative period; B - postoperative neck scar 1 month after surgery).

The view of the postoperative wound of the patient's neck on the second day of the postoperative period is shown in Fig. 4A.

The peculiarity of the postoperative period was the preservation of plugging in the oral cavity for up to 10 days for the reasons described above. Therefore, we carried out repeated tamponing of the oropharynx once every two or three days, focusing on the condition of the tampons.

In the postoperative period, antibacterial, anticoagulant, decongestant, analgesic therapy was conducted. Drugs that reduce salivation were used. The introduction of fluid into the gastrostomy was started on the first day. After the restoration of intestinal motility (it took 48 h according to our observation), the patient was fed with nutrient mixtures and mechanically processed food. The tracheostomy had been removed on the 10th day, after the completion of the period of plugging the oral cavity and regression of signs of laryngeal edema, confirmed instrumentally and clinically. On the 11th day, an X-ray examination of the pharynx with a triombrast solution was conducted - no pathological streaks had been detected. The gastorstomy had been removed on the 14th day. On the 17th day the patient had been discharged in a satisfactory condition. The control examination after 1 month: no complaints from the respiratory and digestive organs. Neck muscles functioning violations were not observed. The view of the postoperative neck scar 1 month after the operation is shown in Fig. 4B.

## 3 Discussion

In myoplasty method the following criteria must be followed: the muscle flap must be of sufficient length and width, so as not to cause excessive tension in the myoplasty area; the flap must be thick enough to avoid necrosis that may cause subsequent infectious complications; when taking the flap, the most sparing operative access should be used, which does not lead to functional and anatomical disorders; the volume of the taken muscle flap must not lead to functional and anatomical disorders [7,8,10].

The most consistent with the specified criteria for myoplasty in this area is myoplasty using the sternocleidomastoid muscle (Musculus sternocleidomastoideus) [7,[14], [15], [16]].

In this patient, the purpose of myoplasty was additional sealing of the pharyngeal suture and myoplasty of the thyroid cartilage injury zone for the purpose of aerostasis. Taking into account the size of the wounds and their anatomical localization, we used the mobilized lower edge of the Musculus sternocleidomastoideus for myoplasty and proposed the method of ladder myoplasty developed by us.

Performing myoplasty with a single flap for two injuries in this case is associated with a high risk of flap tension, which can be complicated by its separation or dysfunction of the remaining part of the muscle. The use of counter myoplasty with strands of the left and right Musculus sternocleidomastoideus may cause additional unjustified surgical injury.

The need for a tracheostomy in this case was necessary to ensure the unobstructed respiratory tract, taking into account the previously described changes. Also to enable packing of the oropharynx, which reduced the contact of the sutures of the pharynx with the contents of the oral cavity (saliva). This protected the pharyngeal suture from the enzymatic effects of saliva and prevented early resorption of the pharyngeal sutures. According to the data of domestic researchers, by the 10th day, persistent fibrin deposits form in the area of  the pharyngeal suture, forming a resistant protective layer from the enzymatic effects of the contents of the oral cavity [6]. Performing a gastrostomy according to Kader allowed to ensure proper nutrition of the patient in the postoperative period and avoid gastroesophageal reflux, which is often observed with prolonged operation of the nasogastric tube.

## 4 Conclusion

The proposed method of ladder myoplasty using Musculus sternocleidomastoideus is unique, proves its high efficiency in unilateral multiple laryngopharyngeal injuries, and can be recommended for wide clinical implementation in such clinical situations.

## Funding

None.

## Ethical approval

This study has been exempted from ethical approval by our institution.

## Consent

Written informed consent was obtained from the patient for publication of this case report and accompanying images. A copy of the written consent is available for review by the Editor-in-Chief of this journal on request.

## Registration of research studies

Not applicable.

## Guarantor

Smolianyk K. M.

## Epigraph

Dedicated to the heroic deeds of Ukrainian medical doctors in the name of life.

## CRediT authorship contribution statement

Igor A. Lurin, study concept and design, data collection, data analysis and interpretation, writing the

paper, artwork editing, bibliographic research

Vitaliy V. Makarov, data collection, data analysis and interpretation, writing the paper, bibliographic

research.

Eduard M. Khoroshun, data collection, data analysis and interpretation, grammar correction.

Volodymyr V. Nehoduiko, data analysis and interpretation, writing the paper, artwork editing.

Sergiy A. Shypilov, data collection, data analysis and interpretation.

Kostiantyn M. Smolianyk, data analysis and interpretation, writing the paper, artwork editing,

visualization.

## Declaration of competing interest

There are no conflicts of interest in the creation of this case report as declared by the authors.
